# An atypical presentation in a child with propionic acidemia? Better think twice!

**DOI:** 10.1002/jmd2.12464

**Published:** 2025-03-12

**Authors:** Tim Burkhardt, Katharine L. Kastor, Stine Christ, Thomas Opladen, Christian Staufner, Stefanie Beck‐Wödl, Tobias Haack, Maja Hempel, Angelika Seitz, Stefan Kölker

**Affiliations:** ^1^ Medical Faculty, Center for Pediatrics and Adolescent Medicine, Division of Pediatric Neurology and Metabolic Medicine Heidelberg University Heidelberg Germany; ^2^ Department of Pediatrics and Adolescent Medicine Marienhaus Klinikum St. Elisabeth Neuwied Neuwied Germany; ^3^ Institute of Medical Genetics and Applied Genomics, Eberhard Karls University Tübingen Tübingen Germany; ^4^ Medical Faculty, Institute of Human Genetics Heidelberg University Heidelberg Germany; ^5^ Medical Faculty, Department of Neuroradiology Heidelberg University Heidelberg Germany

**Keywords:** biotin‐thiamine‐responsive basal ganglia disease, hyperammonemia, lactate acidosis, neurological deterioration, propionic acidemia, propionic aciduria, SLC19A3, thiamine metabolism dysfunction syndrome 2

## Abstract

This report details the case of an infant with confirmed propionic acidemia who presented with progressive neurological deterioration and recurrent episodes of metabolic decompensation with elevated lactate levels, but without hyperammonemia. The child's clinical course and neuroradiological findings increasingly deviated from the known clinical and neuroradiological spectrum of propionic acidemia. A rapid trio exome sequencing identified *SLC19A3*‐related thiamine metabolism dysfunction syndrome 2 as a second genetic disease. The pathomechanisms of both diseases synergize in the impairment of brain energy metabolism, and the associated clinical phenotypes partially overlap, which explains the severe and atypical course of propionic acidemia in the reported case.

**Synopsis**

The search for an additional genetic disorder should be carefully considered in patients with one confirmed diagnosis if the clinical phenotype starts to progress in an “atypical” direction and/or recommended therapy is not as beneficial as expected.

## 1 INTRODUCTION

Propionic acidemia (PA; OMIM #606054) is a rare autosomal recessive disorder caused by an inherited deficiency of the biotin‐dependent dodecameric mitochondrial matrix enzyme propionyl‐CoA carboxylase (PCC; EC 6.4.1.3). Deficient PCC activity results in accumulation of propionyl‐CoA and other metabolites of the propionate oxidation pathway (e.g., propionylcarnitine, 3‐hydroxypropionate, 2‐methylcitrate, and lactate). Propionyl‐CoA and 2‐methylcitrate synergistically impair energy metabolism,^1^, ^2^ and propionyl‐CoA impairs ureagenesis.^3^ As a consequence, individuals with PA have an increased risk of acute metabolic decompensations which are precipitated by catabolic stress and are biochemically characterized by metabolic acidosis, elevated lactate, elevated ketone bodies, and hyperammonemia. PA exhibits phenotypic diversity: The initial disease manifestation commonly affects the brain and later progresses to multiple organ dysfunction, especially cardiomyopathy. In untreated neonates with PA, the disease often progresses from poor feeding and vomiting to hypoglycemia, neurological deterioration with muscular hypotonia, irritability, and lethargy. This could ultimately result in coma or even premature death within a few days. Individuals with late‐onset PA present with a wider range of symptoms with or without apparent metabolic decompensations. The acute management of individuals with PA includes measures for anabolic restoration including acid–base balance along with reduction of toxic metabolites. A low‐protein diet and oral supplementation of levocarnitine are recommended for the long‐term management^4^; the use of coenzyme Q_10_ may be considered.^5^ The described metabolic management does not reliably protect against the progression to multiple organ dysfunction with age. Early liver transplantation is increasingly discussed as a therapeutic option; however, long‐term clinical comparisons to non‐transplanted individuals with PA are needed to systematically evaluate risks and benefits.^4^

## 2 CASE PRESENTATION

We report on an infant with PA who exhibited a history of recurrent episodes of metabolic decompensation and progressive neurological deterioration despite an early diagnosis and metabolic treatment. She was born at term (birthweight: 3.4 kg, 55th percentile) as the second child of consanguineous parents (fourth degree) after uneventful pregnancy and delivery to a family with a history of PA (older sister), α‐thalassemia minor (older sister), and idiopathic epilepsy (mother). Therefore, intravenous glucose (10 mg/kg/d) was administered to prevent a potential neonatal metabolic decompensation. The infant was tested immediately after birth, and the diagnosis of PA was confirmed both biochemically and genetically (homozygous, pathogenic variant in *PCCA*: c.1456C>T:p.Arg486Ter).

At 3 weeks of age, she was admitted to the hospital during an episode of moderate hyperammonemia (146 μmol/L; reference range: 12–53 μmol/L) and elevated lactate (6.7 mmol/L; reference range <1.8 mmol/L), necessitating metabolic emergency treatment. She soon recovered and biochemical results returned to normal. However, a few days later she presented with a brief episode of shrill crying and lip smacking, a sunset phenomenon, and hyperextension of the trunk, which was stopped with a single dose of intravenous phenobarbitone. At this time, plasma ammonium was 74 μmol/L, but lactate peaked at 10 mmol/L. The cofactors thiamine (25 mg/kg/d), biotin (2.5 mg/kg/d), riboflavin (15 mg/kg/d), and coenzyme Q_10_ (10 mg/kg/d) were temporarily added until lactate normalized within 24 h. Cranial and abdominal ultrasound were normal, infectious parameters were negative, and two electroencephalograms did not reveal suggestive electric patterns consistent with seizures. A brain MRI was not performed at this time.

**FIGURE 1:**
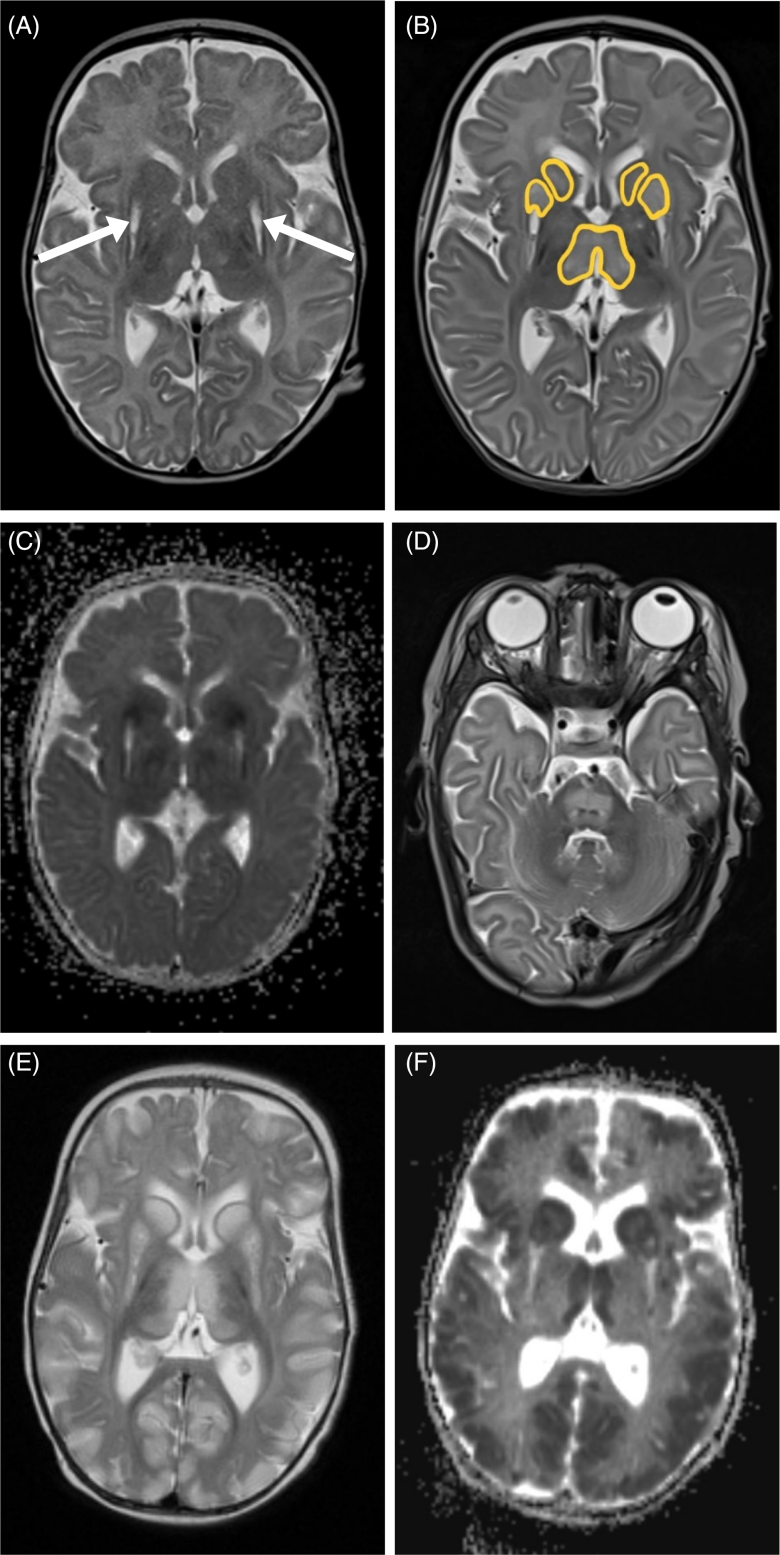
Cranial MRI at three different points of time: A, B through D, E, and F. (A, B, D, E) T2‐weighted images; A ‐ The white arrows indicate the symmetric lesions in both putamina, typically found in individuals with PA; B ‐ New hyperintense lesions in basal ganglia marked in yellow; D ‐ Symmetric pontine hyperintensity; E ‐ New massive, hyperintense lesions involving cerebral cortex. (C, F) Diffusion‐weighted imaging; black areas indicating cytotoxic edema.

Five weeks later, the infant was admitted to the local hospital following a prolonged episode of similar semiology, which was stopped by the administration of midazolam and levetiracetam. Lumbar puncture did not provide evidence to suggest an infectious etiology while cranial magnetic resonance imaging (cMRI) revealed pre‐existing symmetric lesions in both putamina, reminiscent of metabolic stroke (Figure 1A). Lumbar puncture and cMRI were performed under sedation (propofol and midazolam) from which the infant did not wake up but developed severe hypercapnia and respiratory acidosis necessitating mechanical ventilation at the pediatric intensive care unit (PICU). Plasma ammonium and lactate concentrations remained within the normal range. As the coma persisted, another cMRI was performed 3 days later, revealing extensive symmetric lesions in the brainstem, basal ganglia, and occipital and central cerebral cortex (Figure 1B). These neuroradiological findings were not characteristic for PA while newly emerged infratentorial lesions led to the suspicion of central pontine myelinolysis. However, carefully documented blood tests at the PICU did not yield any evidence of electrolyte derangement. This episode resulted in significant neurological sequelae, such as impaired eye fixation, severe muscular hypotonia, conspicuous movement patterns, and failure to thrive. The infant recovered substantially over the next weeks. Since the etiology of the brain lesions was not convincingly explained by PA, a second genetic disease was considered.

Prior to the planned whole exome sequencing (WES), the infant was readmitted to the hospital at 4 months of age due to a SARS‐CoV‐2‐driven episode of neurological deterioration with elevated lactate (9.8 mmol/L) and normal plasma ammonium (56 μmol/L). At that time, cMRI revealed declining pontine lesions, but massive pan‐cortical lesions (Figure 1E), which finally raised the suspicion of *SLC19A3*‐related thiamine metabolism dysfunction syndrome 2 (THMD2, synonym, biotin‐thiamine‐responsive basal ganglia disease; OMIM #607483). In fact, two and a half weeks later, rapid trio exome sequencing identified a homozygous splice site variant (c.980‐2A>G:p.?; variant of uncertain significance) in *SLC19A3* consistent with THMD2. In this case, clinical phenotyping and metabolic profiling were essential to resolve this variant of uncertain significance. Both parents were heterozygous for the familial variant, but the sister's testing was negative. During exome sequencing, pharmacological doses of thiamine (25 mg/kg/d) and biotin (10 mg/kg/d) were introduced into the therapy of the infant. Since then, no further decompensation occurred. In the end, the infant died of an upper respiratory tract infection at the age of 14 months.

## 3 DISCUSSION

In infancy, the immature brain of individuals with PA is particularly vulnerable, resulting in movement disorders due to metabolic stroke and cognitive dysfunction. The first cMRI study in the reported infant revealed pre‐existing symmetric lesions in both putamina (Figure 1A), typically found after metabolic stroke in individuals with PA. The term “metabolic stroke” refers to the acute onset of a central neurological deficit, often associated with a decompensation of the underlying inborn error of metabolism, which cannot be accounted for by hypoxemia or vascular insufficiency.^4^ Several cases of PA with metabolic stroke without metabolic decompensation have been published^6^, ^7^; however, not as early in life as in this child. The timing of the presumed metabolic stroke was initially unclear in this child, since no severe acute metabolic decompensation was observed. Therefore, perinatal stress was assumed as a potential trigger. The following cMRI showed new lesions in the basal ganglia and brainstem (Figure 1B–D), a brain region not characteristically involved in PA.

**FIGURE 2:**
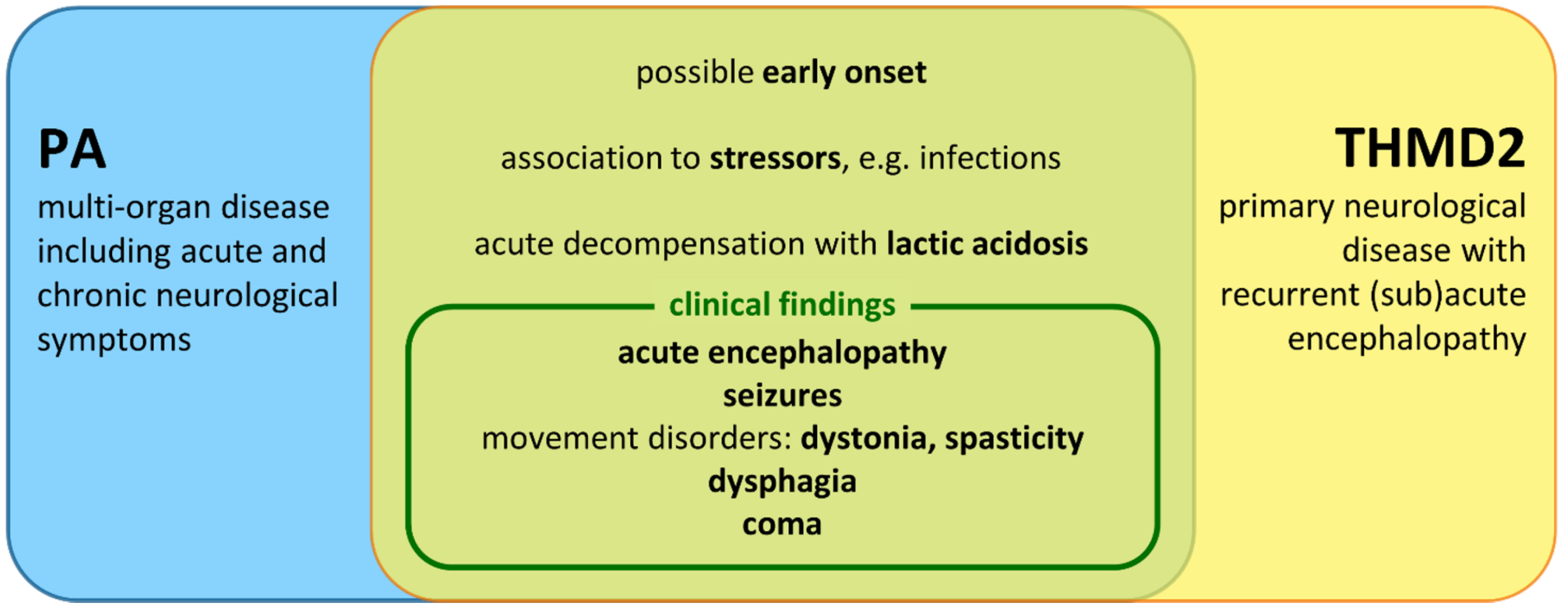
Overlapping clinical phenotypes in PA and THMD2 (green intersection). Both diseases may have an early onset in childhood and are associated to trigger events, for example, febrile illness. Acute metabolic decompensation also leads to lactic acidosis due to its effect on energy metabolism (Figure 3). PA is a multi‐organ disease with acute and chronic neurological symptoms: for example, encephalopathy, seizures, movement disorders, mental retardation, and coma.^4^ THMD2 is a primary neurological disease characterized by recurrent (sub)acute onset of encephalopathy, seizures and attendant neurological symptoms.^6^

**FIGURE 3:**
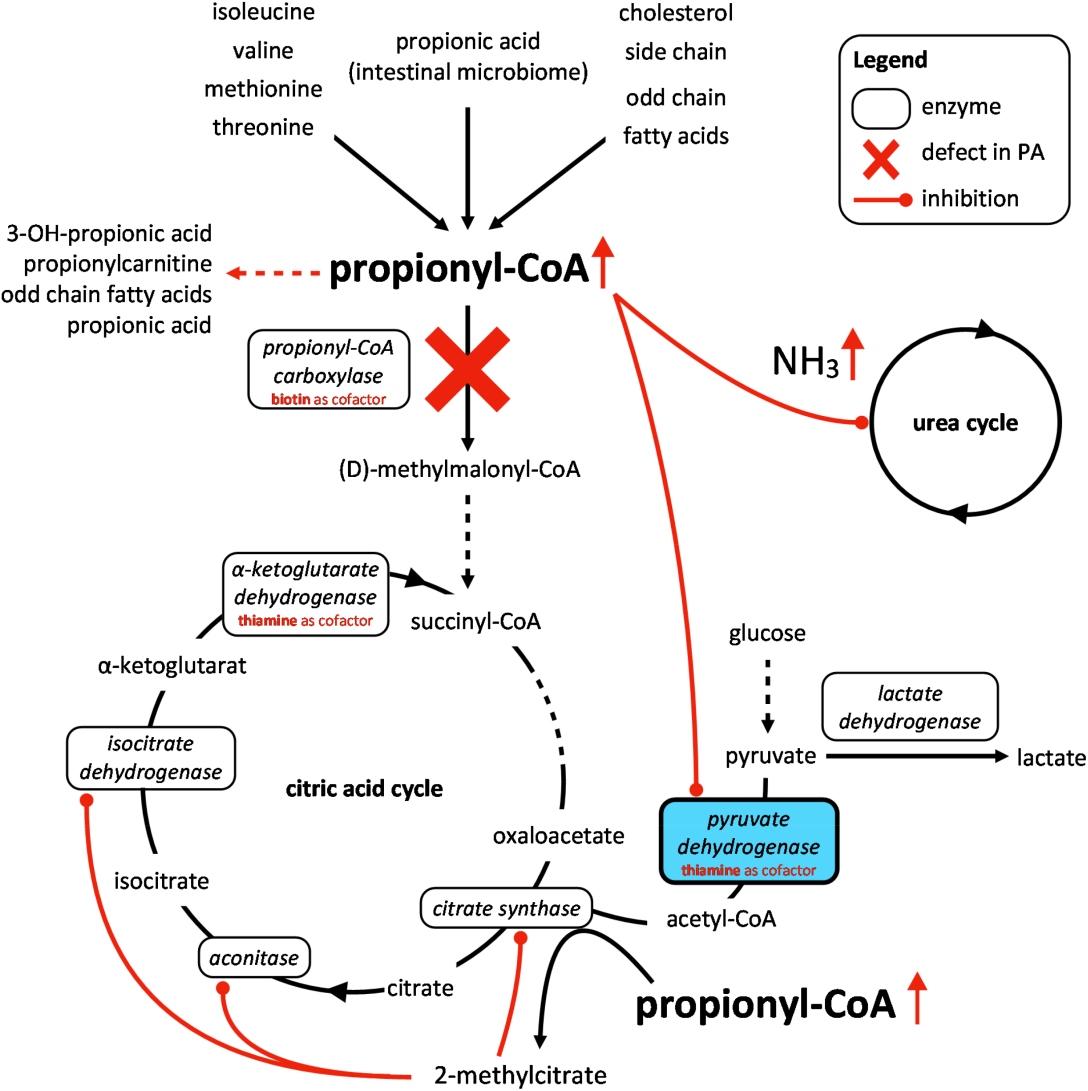
Synergistic pathomechanism of PA and THMD2. The deficiency of the propionyl‐CoA carboxylase in PA results in increased levels of propionyl‐CoA. Propionyl‐CoA itself inhibits the thiamine‐dependent PDHc catalyzing the interface reaction between anaerobic and aerobic glycolysis. Individuals suffering from THMD2 are in higher requirement for thiamine and biotin than can be met by nutrition. The impaired function of pyruvate dehydrogenase leads to elevated lactate in both diseases and reinforces mutually.^9^ In PA, high levels of propionyl‐CoA are proceeded to 2‐methylcitrate by citrate synthase. 2‐methylcitrate then inhibits citrate synthase as well as aconitase and isocitrate dehydrogenase.^1^ Thiamine is a cofactor of another enzyme of the citric acid cycle: α‐ketoglutarate dehydrogenase. PA and THMD2 both lead to an impaired energy metabolism due to their synergistic impact on the pyruvate dehydrogenase and the Krebs cycle. Furthermore, propionyl‐CoA directly inhibits N‐acetylglutamate synthase secondarily leading to hyperammonemia.^3^

In fact, the recurrent episodes of neurological deterioration were associated with transiently elevated plasma lactate levels rather than hyperammonemia. Finally, the combination of cMRI patterns atypical for PA and metabolic derangements without hyperammonemia finally triggered the search for another genetic disease using WES. THMD2 is a rare autosomal recessive neurometabolic disorder characterized by recurrent (sub)acute onset of encephalopathy and basal ganglia lesions. It is caused by bi‐allelic pathogenic variants in *SLC19A3* (2q36.3), which encodes thiamine transporter 2. Individuals with THMD2 are treated with high‐dose thiamine and biotin as early as possible and for life,^8^ which is not used as standard treatment for PA.^4^ The clinical phenotypes (Figure 2) of PA and THMD2 partially overlap especially in terms of encephalopathy and movement disorders. In addition, their pathomechanisms (Figure 3) synergize in impaired function of the pyruvate dehydrogenase complex (PDHc), which explains the diagnostic difficulty and the severe cause of the disease. PDHc is a thiamine‐dependent enzymatic complex which is inhibited by propionyl‐CoA^2^ and forms the bottleneck between anaerobic and aerobic ATP production. Decreased thiamine supply to the brain compartment and elevated mitochondrial propionyl‐CoA result in a substantial impairment of brain energy metabolism, which is further aggravated by acting in concert with propionyl‐CoA‐ and methylcitrate‐induced inhibition of enzymatic complexes of the Krebs cycle. All in all, it results in episodic deterioration and progressive brain lesions even in the absence of apparent catabolic triggers.

The following three aspects delayed the search for a second disease: (1) The infant was known to have PA immediately after birth. Since PA has a broad phenotypic spectrum and many of the initial findings were consistent with this diagnosis or had alternative explanations (e.g., suspected pontine myelinolysis), variations from the characteristic clinical and neuroradiological pattern did raise suspicion only after they progressed to some extent. (2) The mother's history of infantile‐onset epilepsy was another relevant confounding factor, since it provided an alternative explanation for the initial episodes suspecting epileptic seizures.^10^ (3) In PA and THMD2, elevated lactate is a common laboratory finding in both diseases, but prolonged seizure activity can also lead to transiently increased lactate. Finally, the persistent absence of significant hyperammonemic derangements despite progressive brain lesions stimulated the search for another disease.

This case report highlights that the search for an additional genetic disorder should be carefully considered in patients with one confirmed diagnosis if the clinical phenotype starts to progress in an “atypical” direction and/or the recommended therapy is not as beneficial as expected.

## AUTHOR CONTRIBUTIONS

S.K. and T.B. collected the data, drafted the manuscript and prepared the figures. S.K., T.O., and C.S. provided clinical expertise and specialist scientific content. S.B.W., T.H., M.H., S.C., and K.L.K. contributed to data analysis. A.S. contributed decisive neuroradiologic expertise and participated in data editing. All authors revised the manuscript for content and approved the final version.

## FUNDING INFORMATION

This work did not receive funding.

## CONFLICT OF INTEREST STATEMENT

The authors declare no conflicts of interest.

## ETHICS STATEMENT

Ethics approval was not required.

## INFORMED CONSENT

Written informed consent was obtained directly from the parents.

## Data Availability

Additional data are available upon reasonable request of the corresponding author.

## References

[1] Cheema‐Dhadli S , Leznoff CC , Halperin ML . Effect of 2‐methylcitrate on citrate metabolism: implications for the management of patients with propionic acidemia and methylmalonic aciduria. Pediatr Res. 1975;9:905‐908. 127973 10.1203/00006450-197512000-00008

[2] Gregersen N . The specific inhibition of the pyruvate dehydrogenase complex from pig kidney by propionyl‐CoA and isovaleryl‐Co‐A. Biochem Med. 1981;26:20‐27. 7295301 10.1016/0006-2944(81)90026-0

[3] Coude FX , Sweetman L , Nyhan WL . Inhibition by propionyl‐coenzyme A of N‐acetylglutamate synthetase in rat liver mitochondria. A possible explanation for hyperammonemia in propionic and methylmalonic acidemia. J Clin Invest. 1979;64:1544‐1551. 500823 10.1172/JCI109614 PMC371306

[4] Forny P , Hörster F , Ballhausen D , et al. Guidelines for the diagnosis and management of methylmalonic acidaemia and propionic acidaemia: first revision. J Inherit Metab Dis. 2021;44:566‐592. 33595124 10.1002/jimd.12370 PMC8252715

[5] Stanescu S , Belanger‐Quintana A , Fernández‐Felix BM , et al. Plasma CoQ10 status in patients with propionic Acidaemia and possible benefit of treatment with ubiquinol. Antioxidants. 2022;11:1588. 36009307 10.3390/antiox11081588 PMC9405378

[6] Scholl‐Bürgi S , Haberlandt E , Gotwald T , et al. Stroke‐like episodes in propionic acidemia caused by central focal metabolic decompensation. Neuropediatrics. 2009;40:76‐81. 19809936 10.1055/s-0029-1231065

[7] Nyhan WL , Bay C , Beyer EW , et al. Neurologic nonmetabolic presentation of propionic acidemia. Arch Neurol. 1999;56:1143‐1147. 10488817 10.1001/archneur.56.9.1143

[8] Tabarki B , Al‐Hashem A , Alfadhel M , eds. GeneReviews® [Internet]. University of Washington; 2020.

[9] Kölker S , Hoffmann GF . Organoazidurien. Pädiatrie. 2020;5:689‐704.

[10] Kölker S , Valayannopoulos V , Burlina AB , et al. The phenotypic spectrum of organic acidurias and urea cycle disorders. Part 2: the evolving clinical phenotype. J Inherit Metab Dis. 2015;38:1059‐1074. 25875216 10.1007/s10545-015-9840-x

